# Development and Validation of a Radiosensitivity Prediction Model for Lower Grade Glioma Based on Spike-and-Slab Lasso

**DOI:** 10.3389/fonc.2021.701500

**Published:** 2021-07-30

**Authors:** Zixuan Du, Shang Cai, Derui Yan, Huijun Li, Xinyan Zhang, Wei Yang, Jianping Cao, Nengjun Yi, Zaixiang Tang

**Affiliations:** ^1^Department of Biostatistics, School of Public Health, Medical College of Soochow University, Suzhou, China; ^2^Jiangsu Key Laboratory of Preventive and Translational Medicine for Geriatric Diseases, Medical College of Soochow University, Suzhou, China; ^3^Department of Radiotherapy and Oncology, The Second Affiliated Hospital of Soochow University, Suzhou, China; ^4^School of Data Science and Analytics, Kennesaw State University, Kennesaw, GA, United States; ^5^State Key Laboratory of Radiation Medicine and Protection, School of Radiation Medicine and Protection, Collaborative Innovation Center of Radiation Medicine of Jiangsu Higher Education Institutions, Soochow University, Suzhou, China; ^6^Department of Biostatistics, University of Alabama at Birmingham, Birmingham, AL, United States

**Keywords:** lower grade gliomas, radiosensitivity prediction model, radiosensitivity, spike-and-slab lasso, lasso

## Abstract

**Background and Purpose:**

Lower grade glioma (LGG) is one of the leading causes of death world worldwide. We attempted to develop and validate a radiosensitivity model for predicting the survival of lower grade glioma by using spike-and-slab lasso Cox model.

**Methods:**

In this research, differentially expressed genes based on tumor microenvironment was obtained to further analysis. Log-rank test was used to identify genes in patients who received radiotherapy and patients who did not receive radiotherapy, respectively. Then, spike-and-slab lasso was performed to select genes in patients who received radiotherapy. Finally, three genes (INA, LEPREL1 and PTCRA) were included in the model. A radiosensitivity-related risk score model was established based on overall rate of TCGA dataset in patients who received radiotherapy. The model was validated in TCGA dataset that PFS as endpoint and two CGGA datasets that OS as endpoint. A novel nomogram integrated risk score with age and tumor grade was developed to predict the OS of LGG patients.

**Results:**

We developed and verified a radiosensitivity-related risk score model. The radiosensitivity-related risk score is served as an independent prognostic indicator. This radiosensitivity-related risk score model has prognostic prediction ability. Moreover, the nomogram integrated risk score with age and tumor grade was established to perform better for predicting 1, 3, 5-year survival rate.

**Conclusions:**

This model can be used by clinicians and researchers to predict patient’s survival rates and achieve personalized treatment of LGG.

## Introduction

Glioma is the most common malignant brain tumor in adults and one of the leading causes of death world worldwide. Age-adjusted incidence rates for all gliomas range from 4.67 to 5.73 per 100 000 persons (1). According to the Central Brain Tumor Registry of the United States reports, lower grade gliomas (LGG) consist of diffuse low grade and intermediate grade gliomas (World Health Organization grades II and III) (2). In 2016, presence/absence of isocitrate dehydrogenase (IDH) mutation and 1P/19Q codeletion were introduced to classify glioma based on histology and molecular characteristics by WHO (3). Surgical treatment, radiotherapy, chemotherapy, and a combination of radiotherapy and chemotherapy are the main options for the treatment of LGG. Among them, radiotherapy is the main constituent in the combined modality therapy, which has been shown to increase progression-free survival and improve overall survival for LGG patients (4).

However, heterogeneity in radiosensitivity exists among LGG patients. Large retrospective studies of LGG patients in the National Cancer Database (NCDB) have shown that radiotherapy is associated with improved survival outcomes in patients younger than 40 years of age, histological subtypes of astrocytomas, and early high-dose radiotherapy (5). It is desirable to determine radiosensitive LGG patients before incorporating radiotherapy as part of the combined modality therapy. Currently, radiosensitivity of LGG patients can be predicted by O6-methylguanine-DNA methyltransferase (MGMT) promoter methylation and 1p19q codeletion status (6). Moreover, IDH mutation and 1P/19Q codeletion were found to be associated with survival rate and can be used to predict the response to adjuvant therapy.

Even though, the radiosensitivity cannot be fully explained by existing biomarkers. A possible explanation could be provided by researching the tumor microenvironment (TME). TME plays a vital role in the occurrence, progression, and prognosis of tumors. Cancer cells, immune cells, blood vessels, fibroblasts, and other stromal cells make up the TME (7). TME and cancer therapy are complex interplay. Treatment targeted to the TME can increase the likelihood of a good prognosis for patients. Radiotherapy affects tumor blood vessels and immune cells in TME. Specifically, it causes radiation-induced inflammation through damage to endothelial cells and activates immunosuppressive pathways (8). Radiotherapy can shrink the local tumor, but it can also affect distant lesions due to the immunomodulatory effect initiated by the local tumor microenvironment (9). However, radiosensitivity based on TME in LGG has not been systematically discussed.

For LGG, Wen Yin et al. developed and validated an immune-related risk score system based on six hub genes to estimate the overall survival of LGG patients (10). An IDH1-associated immune prognostic signature includes four genes and a nomogram model was established for diffuse LGG (11). Considering the radiosensitivity of the tumor, Yi Cui et al. developed gene signatures by integrating radiosensitivity and immune gene signatures for predicting radiotherapy in breast cancer (12). A retrospective analysis validated 24-gene postoperative radiotherapy outcomes score in prostate cancer (13). But, up to now, a model for predicting the benefit of radiotherapy in LGG has not been established.

With the development of personalized oncology therapy, molecular biomarkers play an important role in the prognosis of LGG. To provide an optimal personalized treatment plan for LGG patients, it is important to find biomarkers and establish a radiosensitivity model based on the tumor microenvironment. Therefore, we attempted to develop and validate a radiosensitivity model for predicting the survival for LGG by using the spike-and-slab lasso Cox model. In summary, our study provided new insights into radiotherapy for LGG.

## Materials and Methods

### Data Sources

**Figure 1:**
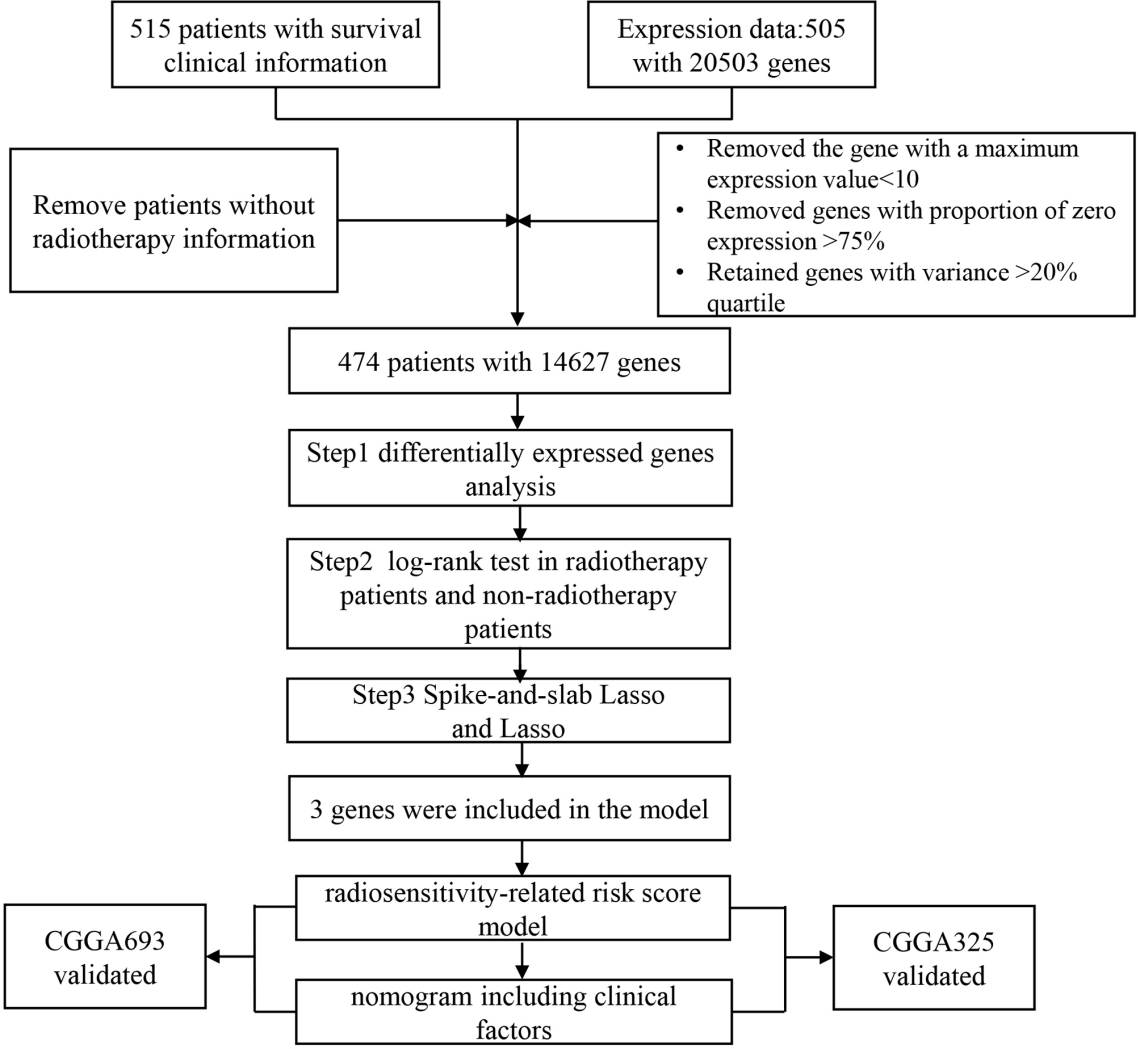
The flow-chart of study design, patient selection and gene selection.

We downloaded 515 LGG patients with clinical and 20503 gene expression datasets from a public database The Cancer Genome Atlas (TCGA, http://cancergenome.nih.gov/) by using the R package TCGA-Assembler (14). Survival information of 534 LGG patients was procured from UCSC Cancer Genomics Browser (https://xenabrowser.net/datapages/) (15). Overall survival (OS) and progression-free survival (PFS) as endpoints. We eliminated the samples without radiotherapy information (n=29) and removed patients with missing survival information(n=3). Considering each gene expression distribution, we also screened genes. The flowchart was summarized in **Figure 1**. Finally, after combining clinical information, RNAseq, and survival information, a final total of finally total of 474 patients with 14627 genes were obtained for the present study. We downloaded gene expression and clinical profiles of 443 LGG patients from CGGA693 dataset (16, 17) and 182 LGG patients from CGGA325 dataset (18, 19) as external validation datasets(http://www.cgga.org.cn/). The cleaned clinical data are summarized in **Supplementary Tables 1**, **2**.

### Differential Expression Analysis and Functional Enrichment Analysis

The Estimation of Stromal and Immune cells in Malignant Tumor tissues using the Expression data (ESTIMATE) tool was used to evaluate the immune and stromal scores for each sample (20). We compared the survival rate between high-scores and low-scores based on the median of each score. Differentially expressed genes (DEGs) analysis was performed by using the *limma* package. The cut-off criteria were adjusted p-value by false discovery rate (FDR) <0.05 and log2 fold-change>1.5. Then, Gene Ontology (GO) and Kyoto Encyclopedia of Genes and Genomes (KEGG) were performed by using the R package clusterProfiler (21).

### Spike-and-Slab Lasso and Lasso

The least absolute shrinkage and selection operator (Lasso) is the commonly used method to select variables. The lasso can select and shrink variables by using the form of the Ɩ_1_-penalty (22). However, the lasso can include many irrelevant predictors and over-shrink large coefficients because of a single penalty.

The spike-and-slab formulation is the core ingredient that can identify promising models (23). Ročková and George developed and applied spike-and-slab Lasso (sslasso) priors to select a variable. spike-and- slab Lasso has a two-point mixture of a Laplace spike distribution (*ѕ*_0_) and a Laplace slab (*ѕ*_1_). *ѕ*_0_ and *ѕ*_1_ are two scale parameters of the spike distribution and slab distribution respectively (24). Therefore, spike-and-slab Lasso has advantages in variable selection and parameter estimation. Recently, Tang et. extended the spike-and-slab lasso framework to generalized linear models and Cox survival models (25, 26). Lasso analysis was performed by using the “glmnet” R package. The model developed by spike-and-slab Lasso was implemented using R package BhGLM (Bayesian hierarchical generalized linear models) (https://github.com/nyiuab/BhGLM) (27).

### Construction and Validation of Radiosensitivity-Related Risk Score

After obtaining the 491 DEGs based on tumor microenvironment, a log-rank test was performed to select genes in patients with radiotherapy and patients who did not receive radiotherapy. We obtained 111 genes for the next analysis. Spike-and-slab Lasso was used to identifying the best prognostic value of these genes. Finally, a radiosensitivity-related risk score was established utilizing spike-and-slab Lasso regression coefficients to multiply the expression values of genes in each patient. We used Kaplan-Meier survival analysis to evaluate the prognostic value of this risk score. Radiosensitive (RS) group and radioresistant (RR) group were defined according to the difference in overall survival rate. The sensitivity and specificity of the model were evaluated by plotting time-dependent receiver operating characteristics (ROC). The radiosensitivity-related risk score was validated in TCGA dataset that PFS as endpoint and two CGGA datasets that OS as the endpoint.

### Development and Validation of the Nomogram

Univariate and multivariate Cox regression analyses were performed to validate whether the risk score has an independent prediction factor. A nomogram to predict the 1-, 3-, and 5-years survival probability were developed according to the results of multivariate Cox analysis. The nomogram was validated in TCGA dataset that PFS as endpoint and two CGGA datasets that OS as the endpoint.

### Analysis Method

All statistical analyses were performed by using the R (4.0.2). The Kaplan-Meier curves were employed to show survival curves. The log-rank test was used to filter radiosensitivity genes based on the tumor microenvironment. Time-dependent ROC curves were plotted by using “timeROC” R package. A nomogram was generated by using the “rms” R package. Infiltration levels for the RS and RR group were quantified by using the bioinformatics tool “CIBERSORT” R package (20). P-value of < 0.05 was considered to be statistically significant. All statistical tests were two-sided.

## Results

### Differentially Expressed Genes Based on Tumor Microenvironment

**Figure 2:**
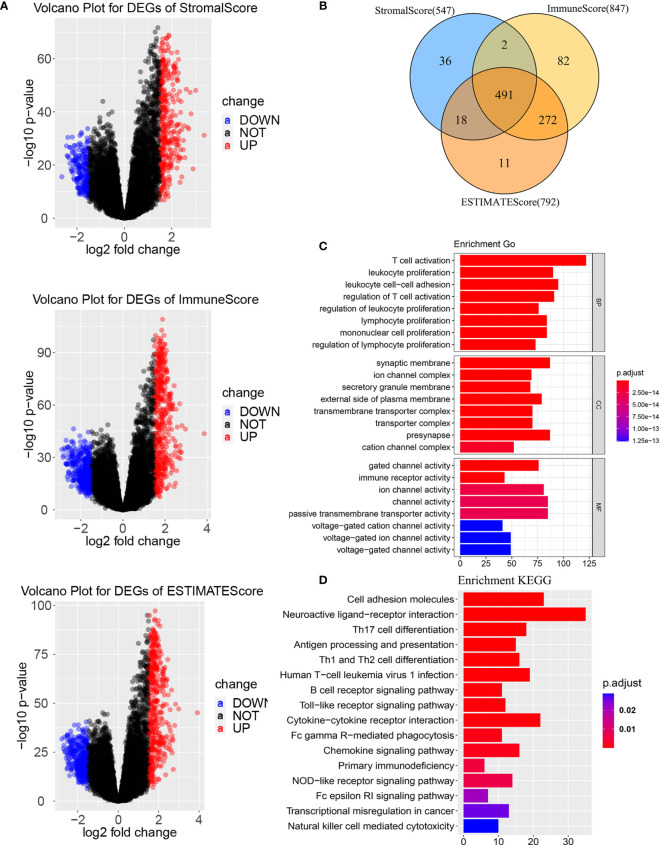
Differentially expressed genes based on tumor microenvironment **(A)** Volcano Plots for DEGs. **(B)** Venn diagram. **(C)** GO enrichment analysis of the DEGs. **(D)** KEGG enrichment analysis of the DEGs. BP, biological process; MF, molecular function; CC, cellular component. GO, Gene Ontology; KEGG, Kyoto Encyclopedia of Genes and Genomes.

To determine the gens of the TME, we calculated the Stromal score, Immune score and ESTIMATE score by using R package “ESTIMATE”. Whole patients were classified into two group according to median score, respectively. As shown in **Supplementary Figure 1**, there was a significant difference between the low stromal-score group and the high stromal-score group (OS: p=0.043, PFS: p=0.025). For the immune-score group, the OS and PFS of the low immune-score group had significantly better than the high immune-score group (OS: p=0.0068, PFS: p=0.020). For the ESTIMATE score, the low ESTIMATE score group had the better OS and PFS than the high ESTIMATE score group (OS: p=0.029, PFS: p=0.039). We also plotted Volcano Plots (**Figure 2A**). Differentially expressed genes (DEGs) analysis between the high- and low-score groups were performed and 491 DEGs were obtained based on the TME(**Figure 2B**). Next, GO analysis demonstrated that the significant biological processes were T cell activation, leukocyte proliferation, and regulation of Tell cell activation (**Figure 2C**). In the KEGG pathway, there were pathways related to the TME, including PI3K-Akt signaling pathway, MAPK signaling pathway, B cell receptor signaling pathway, T cell receptor signaling pathway and PD-L1 expression and PD-1 checkpoint pathway in cancer (**Figure 2D**). These biological functions documented that the DEGs played an important role in TME-related biological procedures in LGG patients.

### Construction of Sensitivity Prediction Model for Radiotherapy

After obtaining differentially expressed genes based on the tumor microenvironment, we performed a log-rank test to identify DEGs associated with radiosensitivity. Whole LGG patients were divided into the high- and low-expression level groups using the median gene expression level as a cutoff point. For radiotherapy patients, the patients in the high- and low-expression group have significant survival differences. However, there was no survival difference between the high- and low- expression level group for non-radiotherapy patients. Ultimately, we obtained 111 genes associated with OS based on TME.

**Figure 3:**
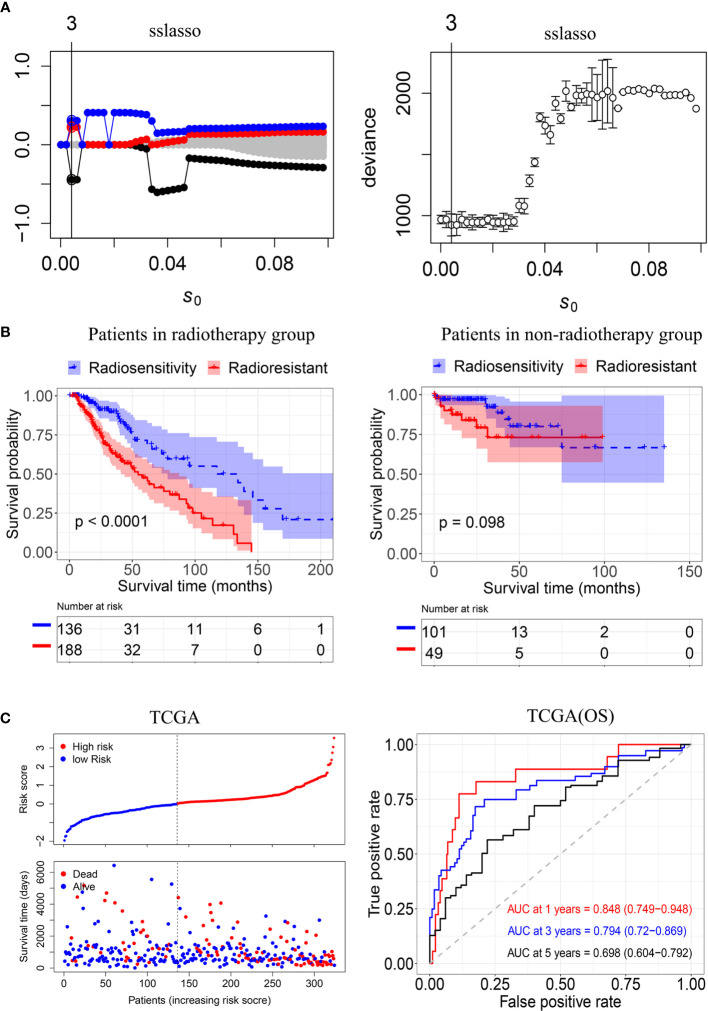
Construction of the radiosensitivity-related risk score model. **(A)** The solution paths and partial log-likelihood profiles of the spike-and-slab model. **(B)** Kaplan–Meier curves for the RS group and RR group in patients with radiotherapy and patients did not receive radiotherapy. RR: radioresistant group. RS: radiosensitive group. **(C)** Risk scores distribution of each patient in the TCGA(OS) and time-dependent ROC curve analysis of the radiosensitivity-related risk score in the TCGA(OS). OS: Overall survival.

To construct a radiosensitivity-related risk score in the TCGA cohort. Spike-and-slab Lasso was used to selecting genes. We fixed the slab scale *ѕ*_1_ to 1 and varied the spike scale *ѕ*_0_ over the grid of values: 0.0001+*k*×0.002, *k* = 0,1,2…,49, leading to 50 models. 10- fold cross-validation was performed to select an optimal model based on the deviance. The minimum value of deviance appears to be 924.330 when the spike scale *ѕ*_0_ is 0.0041 (**Figure 3A**). Therefore, we have chosen the prior scale (0.0041,1) for model fitting and prediction. Finally, three genes were included in the radiosensitivity-related risk score. They are INA (Alpha internexin), LEPREL1 (Leprecan-like 1) and PTCRA (Pre T-cell antigen receptor alpha). And the radiosensitivity-related risk score is the following: Risk score=-0.4442264*INA+0.2253638* LEPREL1+ 0.3067226*PTCRA. Each sample was calculated the radiosensitivity-related risk score. Using the median risk score, patients were divided into high- and low-risk groups. The low-risk group was defined as a radiosensitive group (RS), and the high-risk group was defined as a radioresistant group (RR). The Kaplan–Meier plots indicated that the RS group have a significantly better overall survival than the RR group in the patients who received radiotherapy (p<0.001, **Figure 3B**). There was no difference in overall survival between the RS group and RR group in patients does not receive radiotherapy (p=0.098, **Figure 3B**). Then, we further used ROC analysis to evaluate the predictive ability of radiosensitivity-related risk score model (1-year AUC:0.848 (0.749-0.948); 3-years AUC:0.794 (0.720-0.869); 5-years AUC:0.698 (0.604-0.792), **Figure 3C**).

We also fitted the model by the lasso approach and performed 10-fold cross-validation as a comparison. However, no genes were screened by using lasso (**Supplementary Figure 2** **)**.

### Validation of Radiosensitivity Model in Validation Sets

**Figure 4:**
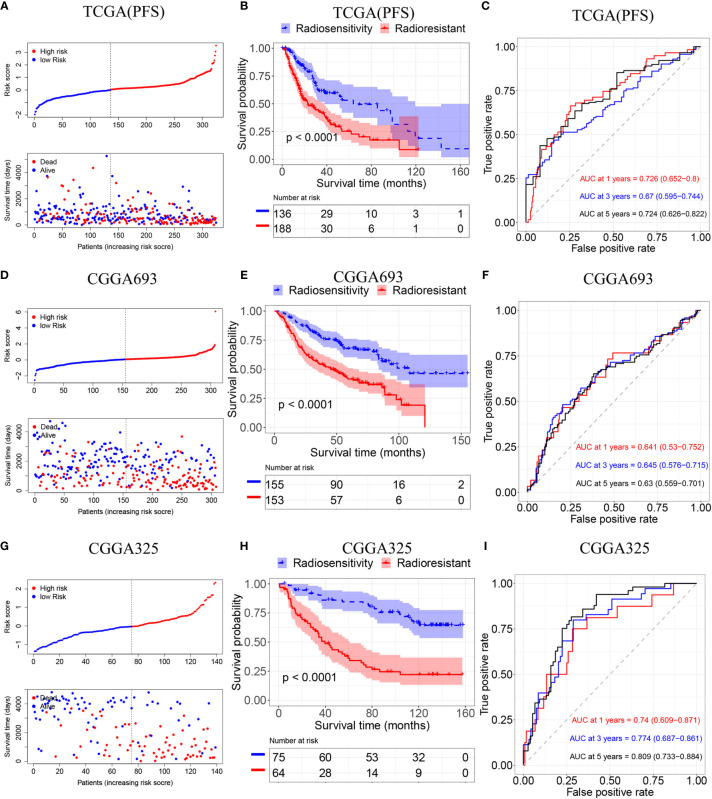
Validation of the radiosensitivity-related risk score model. **(A)** Risk scores distribution of each patient in the TCGA(PFS). **(B)** Kaplan–Meier curves for the RS group and RR group in patients with radiotherapy from TCGA(PFS). **(C)**Time-dependent ROC curve analysis of the radiosensitivity-related risk score in the TCGA(PFS). **(D)** Risk scores distribution of each patient in the CGGA693. **(E)** Kaplan–Meier curves for the RS group and RR group in patients with radiotherapy from CGGA693. **(F)**Time-dependent ROC curve analysis of the radiosensitivity-related risk score in the CGGA693. **(G)** Risk scores distribution of each patient in the CGGA325. **(H)** Kaplan–Meier curves for the RS group and RR group in patients with radiotherapy from CGGA325. **(I)**Time-dependent ROC curve analysis of the radiosensitivity-related risk score in the CGGA325.

To validate the radiosensitivity-related risk score constructed in the TCGA cohort, we applied the risk score formula to PFS outcome in TCGA, CGGA693 and CGGA 325 datasets, respectively **(** **Figure 4** **)**. Each patient has calculated the risk score and divided into RS group and RR group according to median risk score. The Kaplan–Meier analysis showed patients in RS group had a better prognosis while patients in RR group had unfavorable outcomes in radiotherapy patients (TCGA PFI: p<0.001, CGGA693: p<0.001, CGGA325: p<0.001 **Figures 4B, E, H** **)**. ROC curve was used to evaluate the predictive accuracy for 1-, 3-, and 5-year survival. AUC values revealed the high predictive value of the radiosensitivity-related risk score for LGG patients. (TCGA PFI:1-year AUC:0.726 (0.652-0.800); 3-years AUC:0.670 (0.595-0.744); 5-years AUC:0.724(0.626-0.822). CGGA693:1-year AUC:0.641(0.530-0.752); 3-years AUC:0.645(0.576-0.715); 5-years AUC:0.630(0.559-0.701). CGGA325:1-year AUC:0.740(0.609-0.871); 3-years AUC:0.774 (0.687-0.861); 5-years AUC:0.809 (0.733-0.884). **Figures 4C, F, I** **)**.

### The Radiosensitivity-Related Risk Score Is an Independent Prognostic Indicator

**Figure 5:**
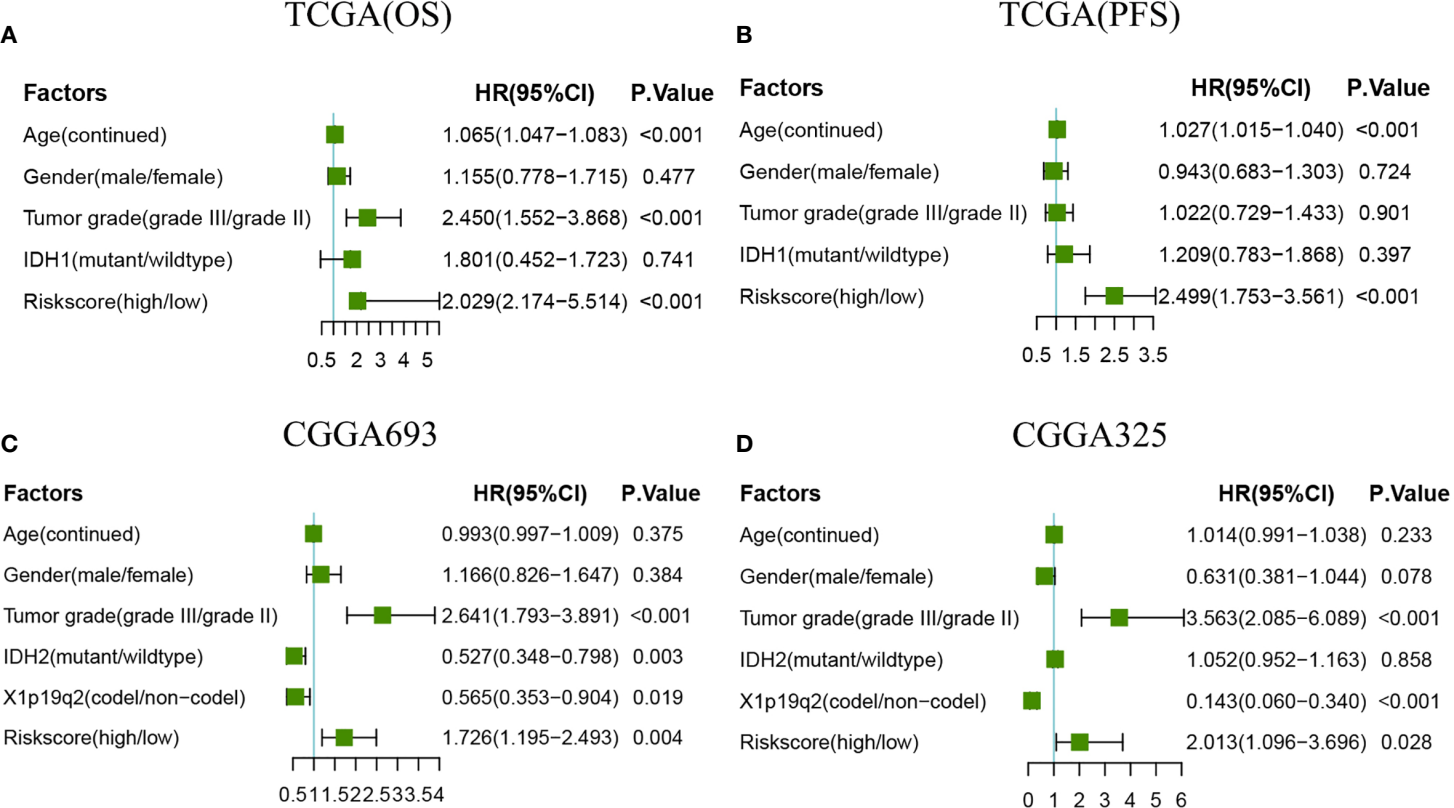
Forest plots of multivariate Cox regression. **(A)** Forest plots of multivariate Cox regression in TCGA(OS). **(B)** Forest plots of multivariate Cox regression in TCGA(PFS). **(C)** Forest plots of multivariate Cox regression in CGGA693. **(D)** Forest plots of multivariate Cox regression in CGGA325.

Univariate and multivariate Cox regression was used to examine whether the radiosensitivity-related risk score was an independent prognostic factor. As demonstrated in **Supplementary Figure 3**. The univariate analysis showed that the age (HR:1.055, 95%CI:1.039-1.071, p<0.001), tumor grade (HR:2.630,95%CI:1.687-4.101, p=0.004) and risk score (HR:2.864, 95%CI:1.822-4.503, p<0.001) were significantly associated with OS. After adjusted clinical factors such as age, gender, tumor grade, race, IDH1, the multivariate Cox regression result showed that radiosensitivity-related risk score was an independent prognostic factor for LGG patients. When the OS as an endpoint, the HR was 3.657 (95%CI:2.171-6.160, p<0.001, **Figure 5A**). When the PFS as an endpoint, the HR was 2.522(HR: 2.522, 95%CI:1.627-3.908, p<0.001, **Figure 5B**). In CGGA datasets, we adjusted clinical factors such as age, gender, tumor grade, race, IDH2, and X1p19q2, the multivariate Cox regression results also demonstrated that radiosensitivity-related risk score was an independent prognostic factor for LGG (CGGA693: HR:1.726, 95%CI: 1.195-2.493, p=0.004. CGGA325: HR: 2.013, 95%CI: 1.096-3.696, p=0.028. **Figures 5C, D** **)**.

### Construction and Validation of Nomogram

**Figure 6:**
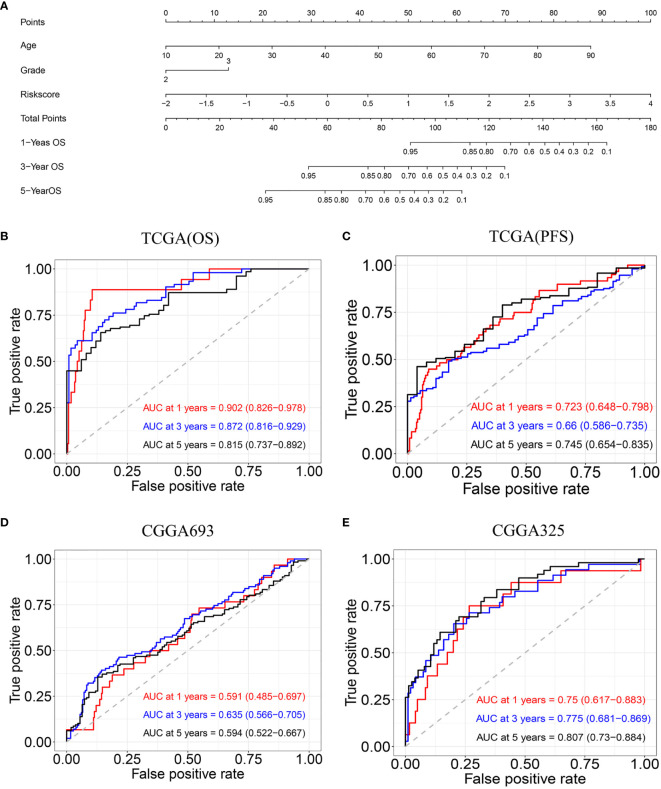
Construction and validation of nomogram model. **(A)** Nomogram model for predicting the probability of 1-, 3-, and 5-year OS in LGG. **(B)** Time-dependent ROC curve analyses of the nomogram model in the TCGA(OS). **(C)** Time-dependent ROC curve analyses of the nomogram model in the TCGA(PFS). **(D)** Time-dependent ROC curve analyses of the nomogram model in the CGGA693. **(E)** Time-dependent ROC curve analyses of the nomogram model in the CGGA325.

The univariate and multivariate Cox regression analyses indicated that age, tumor grade, and risk score are correlated with OS. A nomogram containing two clinical factors (age and tumor grade) and risk score was developed to predict OS rate for LGG patients (**Figure 6A**). From the results of ROC analysis in **Figure 6B**, the AUCs of nomogram at 1-, 3-, 5-year was 0.902, 0.872, 0.815, respectively, which was higher than a model with a radiosensitivity-related risk score. **Figure 6C** demonstrated that the AUCs of nomogram at 1-, 3-, 5-year was 0.723, 0.660 and 0.745 for TCGA(PFS). We also used two CGGA datasets to verify a nomogram. **Figure 6D** demonstrated that the AUCs of nomogram at 1-, 3-, 5-year was 0.591(95%CI:0.485-0.697), 0.635 (95%CI:0.566-0.705) and 0.594 (95%CI:0.522-0.667) for CGGA693. **Figure 6E** demonstrated that the AUCs of nomogram at 1-, 3-, 5-year was 0.750 (95%CI:0.617-0.883), 0.775(95%CI:0.681-0.869) and 0.807 (95%CI:0.730-0.784) for CGGA325.The risk score for the prognostic model displayed superior predictive performance compared with the nomogram in the CGGA325.

### Infiltration Levels for RS and RR Group

**Figure 7:**
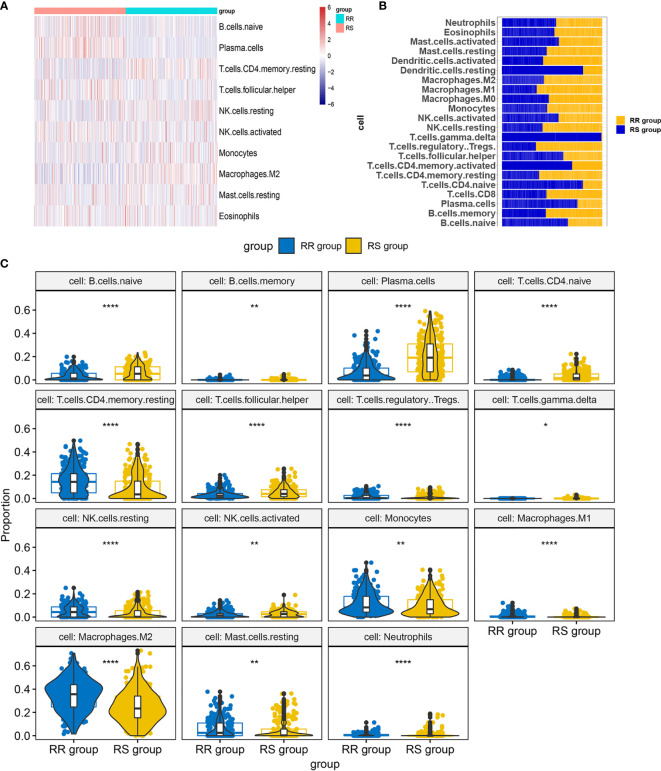
Infiltration levels for RS and RR group. **(A)** Heat map of infiltration cells in RS and RR group. **(B)** Bar graphs of mean percentage of immune cells in RS and RR group. **(C)** Violin plots illustrating the relative proportions of the 15 TIICs exhibiting significantly different infiltrating degree in RS and RR group. RR, radioresistant group. RS, radiosensitive group. *p < 0.05, **p < 0.01, ***p < 0.001, ****p < 0.0001.

We performed infiltration levels for the RS group and RR group in LGG by employing the LM22 signature. In the process of plotting the heat map **(** **Figure 7A** **)**, we removed zero abundance immune cells from more than half of the samples. Next, we estimated mean fractions of immune cells in the RS and RR group **(** **Figure 7B** **)**. Tumor samples in the RS group shown more dendritic cells resting, T cells gamma delta and T cells CD4 naïve than the RR group. 15 tumor-infiltrating immune cells exhibit significantly different relative proportions between the RS and RR group **Figure 7C**.

## Discussion

Treatments of LGG include radiotherapy, chemotherapy and surgery. Fan Wu et. identified immune-related subtypes to select optimally patients suffering from LGG responsive to immunotherapy (28). A retrospective study suggested MRI feature that cyst formation on preoperative MR images was used to predict a favorable prognosis in patients with LGG (29). In a study of more than 1, 0000 people with LGG, researchers noted radiotherapy improved survival outcomes (5). However, the optimal radiotherapy for a particular patient based on individual symptoms and the risk of treatment-induced toxicity remains unclear. Advances have been made in biomarkers that predict response to treatment. Despite the beneficial effects of radiotherapy in LGG patients, this treatment has some significant side effects that should not be disregarded. Therefore, it is important to select biomarkers that can be used to screen radiosensitivity patients.

In this study, we obtained differentially expressed genes based on tumor microenvironment by calculating Stromal score, Immune Score and ESTIMATE Score. Then, we obtained DEGs and performed GO and KEGG. The log-rank test was used to identify genes associated in patients who received radiotherapy and patients who did not receive radiotherapy, respectively. Spike-and-slab Lasso was used to selecting genes. Finally, three genes (INA, LEPREL1, and PTCRA) are included in the model. A radiosensitivity-related risk score model was established based on the overall rate of TCGA dataset in patients who received radiotherapy. And we validated this model with TCGA dataset and two CGGA datasets. This radiosensitivity-related risk score model has prognostic prediction ability and is an independent prognostic indicator. A novel nomogram integrated risk score with age and tumor grade was developed to predict the OS of LGG patients. The nomogram was also validated in two CGGA datasets. According to the radiosensitivity-related risk score and nomogram, clinicians can able to identify a group of patients who are better benefit from radiotherapy and then can predict the 1-, 3-, and 5-years OS of LGG.

The three genes included in our radiosensitivity-related risk score model were INA, LEPREL1, and PTCRA. The INA gene encodes an intermediate filament involved in neurogenesis. α- is the fourth subunit of neurofilaments in the adult central nervous system (30). INA is overexpressed mostly in oligodendroglia phenotype gliomas and correlated with better PFS and OS (31). INA expression on immunohistochemistry in anaplastic gliomas showed a significant positive correlation with 1p/19q codeletion and can replace to some extent 1p/19q (32, 33). INA gene methylation is associated with the progression of colon adenoma (34)and gastroenteropancreatic neuroendocrine neoplasms (35). LEPREL1 similarity to the Leprecan family of proteoglycans and as a 3.4 kb transcript encoding an 80 kDa protein (36). Many pieces of evidence shown that LEPREL1 was associated with ophthalmic diseases such as high myopia and lyens subluxation (37–39). For cancer, LEPREL1 may be a potential tumor suppressor gene by inhibiting HCC cell proliferation (40). LEPREL1 was methylation inactivation of tumor suppressor gene and involved in the pathogenesis of breast cancer (41). PTCRA participates in cancer-related signaling pathways. Research has shown that variation of PTCRA may be related to the prognosis of patients with chronic myelogenous leukemia (42). Unfortunately, we did not find a correlation between PTCRA gene and LGG.

There is growing evidence that the identification of prognostic factors is important for the optimal treatment of LGG patients. Han Sang Kim et al. used the NCI-60 cancer cell line to identify 31-gene signature of radiosensitivity from four different microarrays (43). This signature was verified in breast cancer (44), and low-grade glioma (45). Gene signatures have been successfully used in various cancer types to develop prognostic and predictive models that benefit patients. We developed a radiosensitivity-related risk score model to predict the benefit of radiotherapy in LGG. This study showed that in independent validation cohorts that radiosensitivity patients had significant survival benefits from radiotherapy, whereas there was no difference between RS group and RR group in patients who did not receive radiotherapy. Two studies researched prediction of radiotherapy in prostate cancer (13)and breast cancer (12). They matched patients in the entire cohort and each biomarker-defined group, respectively. In prostate cancer, researchers compared the radiotherapy patients and no radiotherapy patients in high- and low-score. Recently, Xing Chen et al. developed and validated a six gene signature for breast cancer radiotherapy (46). In this article, they used the Kaplan-Meier curve to compare high- and low- score in radiotherapy group. In our study, the definition of radiosensitivity is that patients receiving radiotherapy, this subgroup patients obtained significantly more survival benefit than patients in another subgroup. Moreover, there were no differences between the two subgroups in patients who did not receive radiotherapy.

Selecting genes induced in the model plays an important role in establishing a prediction model. Researchers usually screen genes by using Cox regression analysis, Lasso Cox regression method, random forest algorithm and other methods. In our study, Spike-and-slab Lasso and Lasso were used to selecting genes. Results have shown that three genes were selected by using spike-and-slab Lasso. However, we were unable to screen for the gene with Lasso. Spike-and-slab Lasso can shrink many coefficients exactly to zero and select variables similar to the lasso. Spike-and-slab Lasso has the advantage of diminishing the estimation bias of Lasso by yielding weak shrinkage on important predictors and strong shrinkage on irrelevant predictors (25). The spike-and-slab Lasso method to select has been applied successfully in LGG real data. Our results demonstrated that advantages of spike-and-slab Lasso in screening variables compared with Lasso.

We developed and verified a radiosensitivity-related risk score model. Next, we performed ROC analysis to compare the predictive ability of a risk score model. We compared the radiosensitivity-related risk score model and nomogram, the results showed that the AUC of the nomogram was not significantly improved compared with a radiosensitivity-related risk score model.

Jun Su et al. constructed a prognostic risk score model (Model 1) based on eight TME-related genes using co-expression network analysis (WGCNA) and lasso (47). This model had potential value for predicting the sensitivity of LGG patients to radio- and chemotherapy. We both obtained the immune score and the stromal score by ESTIMATE algorithm. However, we obtained the TME related gene by using DEGs instead of WGCNA. We used the sslasso method to screen genes and developed the radiosensitivity prediction model in patients who received radiotherapy. Jun Su et al. constructed a prognostic risk score model in whole patients. Wen Jing Zeng et al. constructed a survival risk score system (Model 2) based identify prognostic genes associated with promoter methylation by using Cox proportional hazards regression analysis (48). This three-gene signature was validated in CGGA and performed stratified survival analysis. However. this model was not applied to radiosensitivity. Next, we compared these two models in patients who received radiotherapy and patients who not received radiotherapy. As shown in **Supplementary Figure 4**, Kaplan-Meier curves demonstrated that low-risk group had longer OS than high-risk group both in patients who underwent radiotherapy and patients who were not undergoing radiotherapy. Our model took into account not only people who received radiotherapy but also people who did not receive radiotherapy. Kaplan-Meier plots indicated that the RS group have a significantly better overall survival than the RR group in the patients who received radiotherapy (p<0.001, **Figure 3B** **)**. There was no difference in overall survival between the RS group and RR group in patients does not receive radiotherapy (p=0.098, **Figure 3B**). Therefore, our model has better potential to identify RS and RR groups.

Our study provides new insights into the radiotherapy therapies for LGG. The main strength of this study is the method of selecting genes. We applied spike-and-slab Lasso to select genes different from Cox regression and Lasso. The radiosensitivity-related model can identify patients most likely to benefit from radiotherapy. However, a limitation of our study is that this is a retrospective study, and the models should be further confirmed by prospective studies.

In conclusion, the radiosensitivity-related score is an independent prognostic indicator. Patients with LGG can be divided into RS and RR groups. The patients in the RS group are more likely to benefit from radiotherapy. This model can be used by clinicians and researchers to predict patient’s survival rates and achieve personalized treatment of LGG.

## Data Availability Statement

The original contributions presented in the study are included in the article/**Supplementary Material**. Further inquiries can be directed to the corresponding author.

## Author Contributions

Study conception and design: ZD, SC, NY, and ZT. Data collection and clean: ZD, DY, SC, WY, and XZ. Real data analysis and interpretation: ZD, HL, WY, NY, and JC. Drafting of the manuscript: ZD, HL, DY, JC, and XZ. All authors contributed to the article and approved the submitted version.

## Funding

This work was supported in part by the National Natural Science Foundation of China (81773541), funded from the Priority Academic Program Development of Jiangsu Higher Education Institutions at Soochow University, the State Key Laboratory of Radiation Medicine and Protection (GZK1201919) to ZXT, National Natural Science Foundation of China (81872552, U1967220) to JPC. A project by the Second Affiliated Hospital of Soochow University (XKTJ-RC202007), Scientific Research Program for Young Talents of China National Nuclear Corporation (51003), Suzhou Science and Education Project (KJXW2017010), the Natural Science Foundation of Jiangsu Province (BK20180195), the National Natural Science Foundation of China (81902715) to SC The funding body did not play any roles in the design of the study and collection, analysis, and interpretation of data and in writing the manuscript. This work was supported by the National Natural Science Foundation of China (31870844, 31570851) and A Project Funded by the Priority Academic Program Development of Jiangsu Higher Education Institutions (PAPD) to WY.

## Conflict of Interest

The authors declare that the research was conducted in the absence of any commercial or financial relationships that could be construed as a potential conflict of interest.

## Publisher’s Note

All claims expressed in this article are solely those of the authors and do not necessarily represent those of their affiliated organizations, or those of the publisher, the editors and the reviewers. Any product that may be evaluated in this article, or claim that may be made by its manufacturer, is not guaranteed or endorsed by the publisher.

## Supplementary Material

Supplementary Figure 1 Survival curves plot for stromal score, immune score and ESTIMATE score.

Supplementary Figure 2 Lasso Cox analysis.

Supplementary Figure 3 Forest plots of univariate Cox regression. **(A)** Forest plots of univariate Cox regression in TCGA(OS). **(B)** Forest plots of univariate Cox regression in TCGA(PFS). **(C)** Forest plots of univariate Cox regression in CGGA693. **(D)** Forest plots of univariate Cox regression in CGGA325.

Supplementary Figure 4 **(A)**Kaplan–Meier curves for the low riskscore group and high riskscore group in patients with radiotherapy and patients did not receive radiotherapy in model1. **(B)**Kaplan–Meier curves for the low riskscore group and high riskscore group in patients with radiotherapy and patients did not receive radiotherapy in model2.

Supplementary Table 1 Basic patient characteristics in TCGA dataset.

Supplementary Table 2 Basic patient characteristics in CGGA693 and CGGA325 datasets.
