# Supplementary figures and images for: Development and Validation of a Radiosensitivity Prediction Model for Lower Grade Glioma Based on Spike-and-Slab Lasso
Source: Front Oncol. 2021 Jul 30;11:701500. doi: 10.3389/fonc.2021.701500 (PMC8363254; doi:10.3389/fonc.2021.701500)

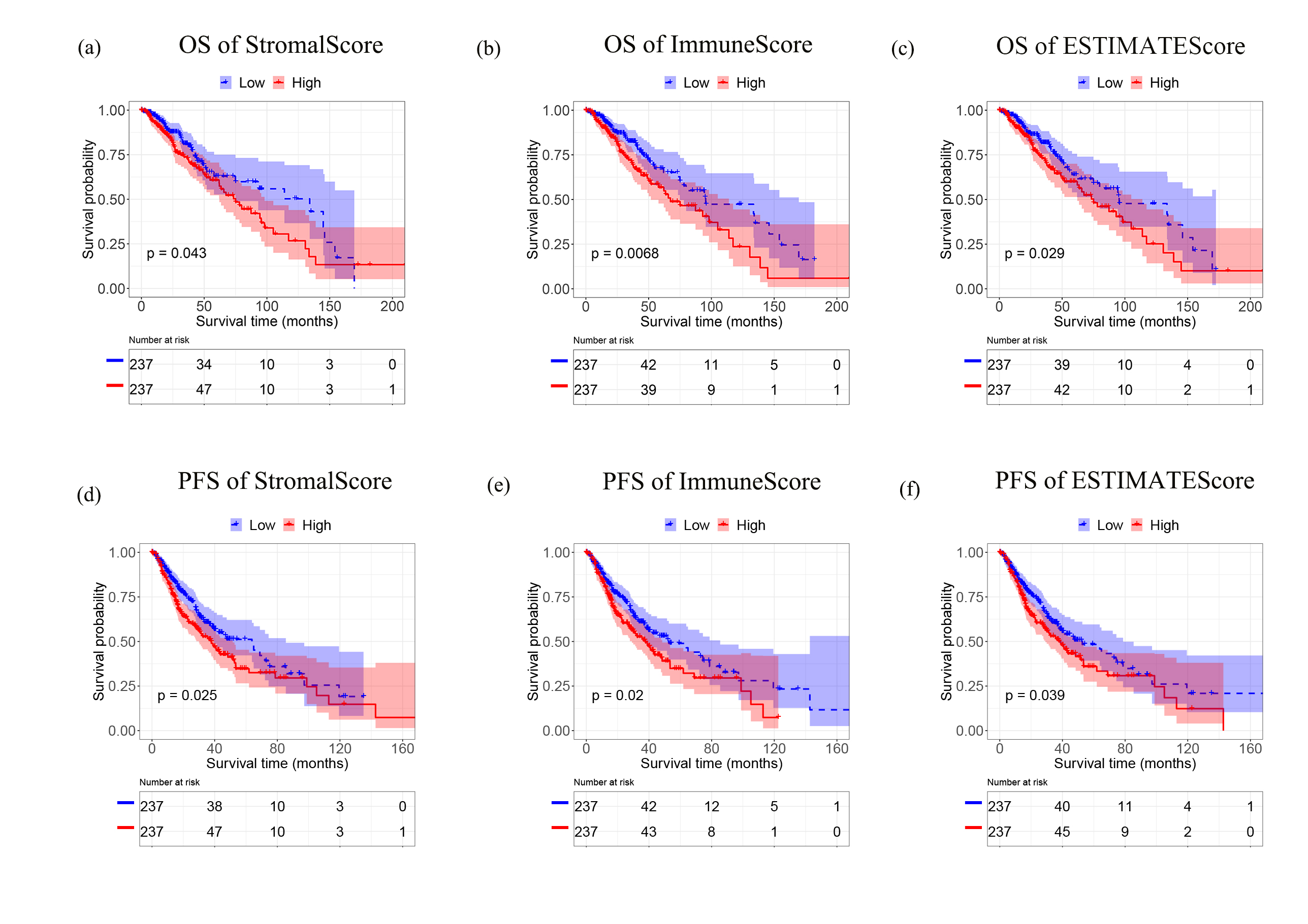

Supplement: Supplementary Figure 1 — Survival curves plot for stromal score, immune score and ESTIMATE score. [file Image_1.jpeg]

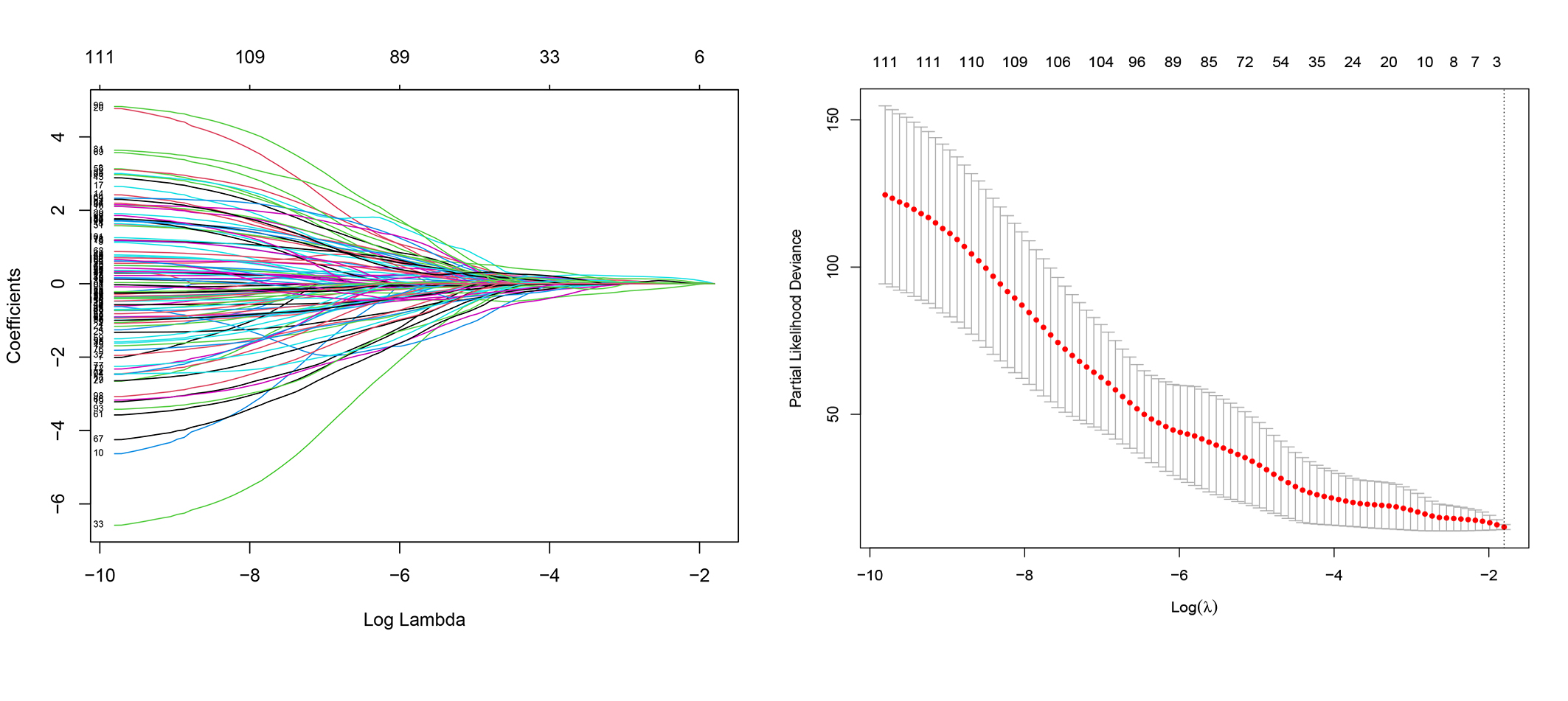

Supplement: Supplementary Figure 2 — Lasso Cox analysis. [file Image_2.jpeg]

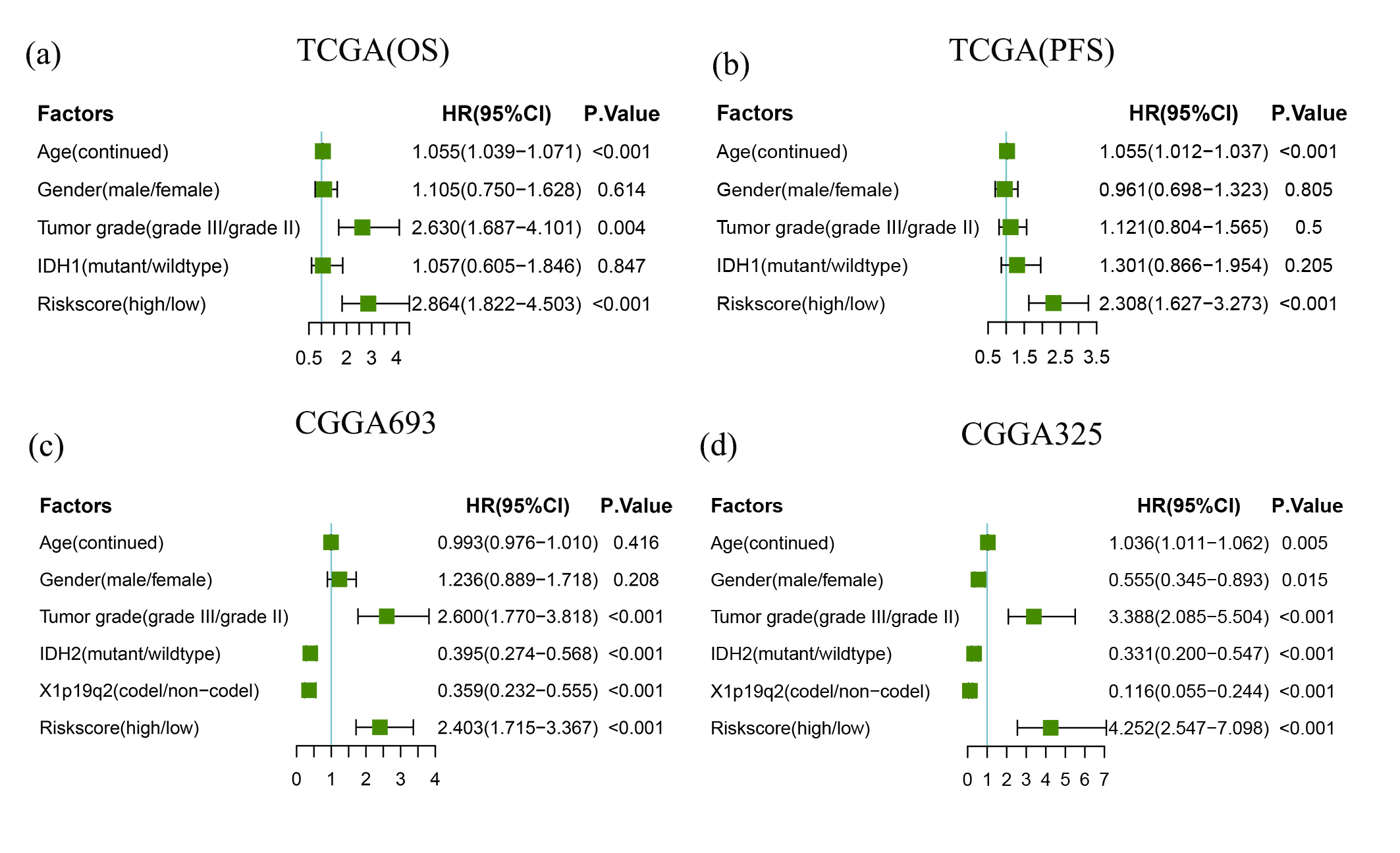

Supplement: Supplementary Figure 3 — Forest plots of univariate Cox regression. (A) Forest plots of univariate Cox regression in TCGA(OS). (B) Forest plots of univariate Cox regression in TCGA(PFS). (C) Forest plots of univariate Cox regression in CGGA693. (D) Forest plots of univariate Cox regression in CGGA325. [file Image_3.jpeg]

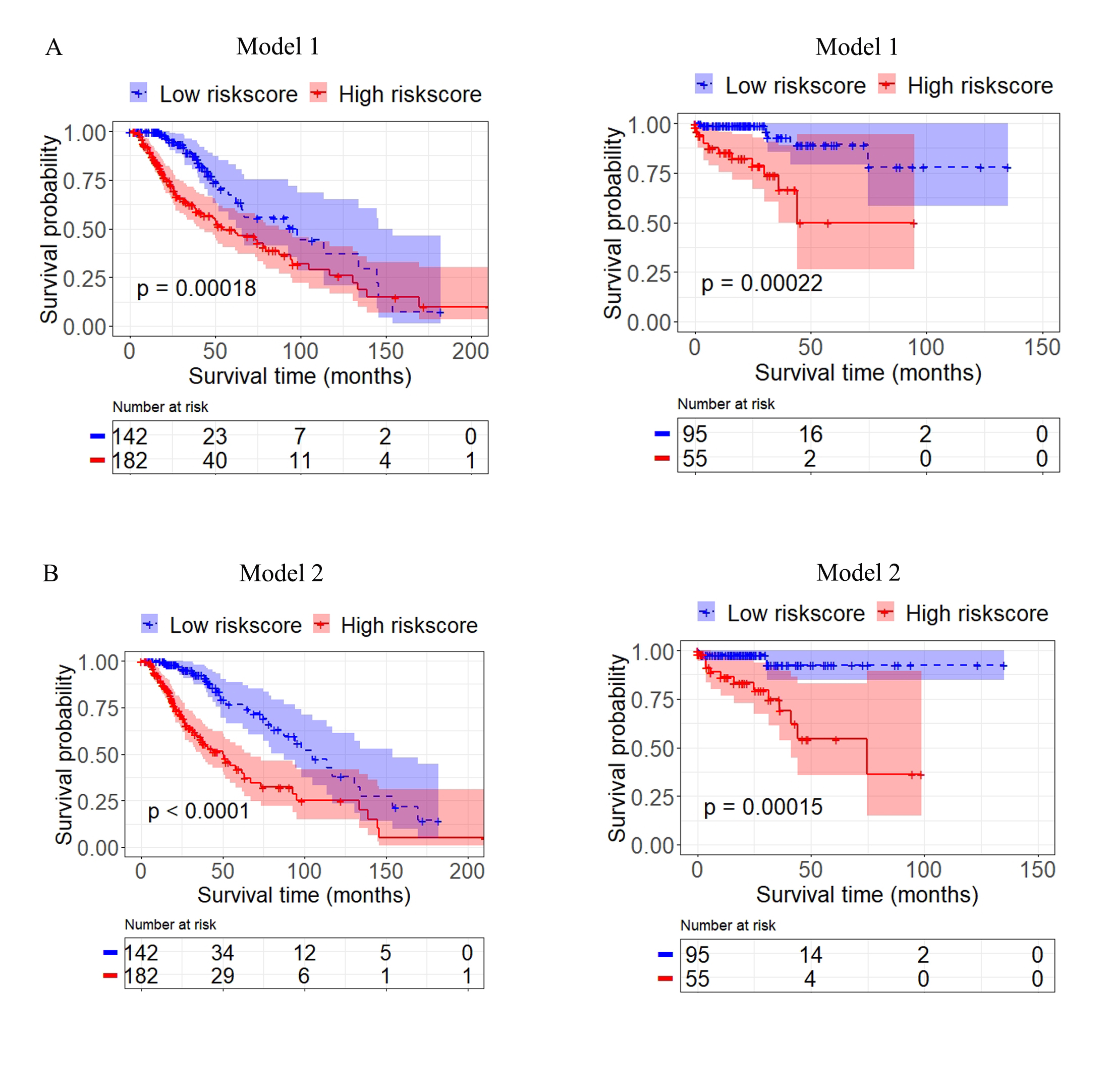

Supplement: Supplementary Figure 4 — (A)Kaplan–Meier curves for the low riskscore group and high riskscore group in patients with radiotherapy and patients did not receive radiotherapy in model1. (B)Kaplan–Meier curves for the low riskscore group and high riskscore group in patients with radiotherapy and patients did not receive radiotherapy in model2. [file Image_4.jpeg]
